# Neotypification of *Clytostoma sciuripabulum* Hovel.

**DOI:** 10.3897/phytokeys.78.10649

**Published:** 2017-03-22

**Authors:** Maria M. Arbo

**Affiliations:** 1 Instituto de Botánica del Nordeste, Herbario CTES, C.C. 209, 3400 Corrientes, Argentina

**Keywords:** Bignoniaceae, Southamerica

## Abstract

A neotype is designated for *Clytostoma sciuripabulum* the basionym of *Bignonia sciuripabulum*, the presently accepted name of the species.

## Introduction

Revision of the species of Bignoniaceae for ‘Flora Argentina’ identified the need for a new typification for the basionym of *Bignonia sciuripabulum.* This is a South American species with a wide distribution, occurring in Colombia, Venezuela, Guyana, Brazil, Ecuador, Peru, Bolivia, Paraguay and Northern Argentina (Lohmann and Taylor 2014: 422).

## Material and methods

Specimens (digital images) kept at K and P were examined on JSTOR Global Plants 2016). Some digital images were obtained from the source herbaria: C and S. The specimens were carefully analysed taking into account the protologues. The articles cited through the text follow the International Code of Nomenclature (ICN), Melbourne Code (McNeill et al. 2012). Herbaria acronyms follow Thiers (2017).

## Historical remarks and typification

The specific epithet “*sciuripabulum*” was used for the first time by K. Schumann (1894: 224) who published the binomial *Arrabidaea sciuripabulum* (Bureau) K.Sch. This name (a *nomen nudum*, see Art. 38.2 Ex.1) is not valid since it has no diagnosis, it is mentioned in a paragraph about the genus *Cydista* which states that the species is related to *Cydista difficilis*.

Two years later, Bureau and K. Schumann (1896: 149) published the name *Clytostoma sciuripabulum* with a detailed diagnosis and description. The only gathering mentioned was *J.C. de Mello 22*, collected in Brasilia, Sao Paulo, prope Campinas. Nine specimens of this collection have been located, kept in C, K, P and S, with different dates or not dated (Table 1). According to Art. 8.2, these specimens are syntypes.

**Figure 1.**
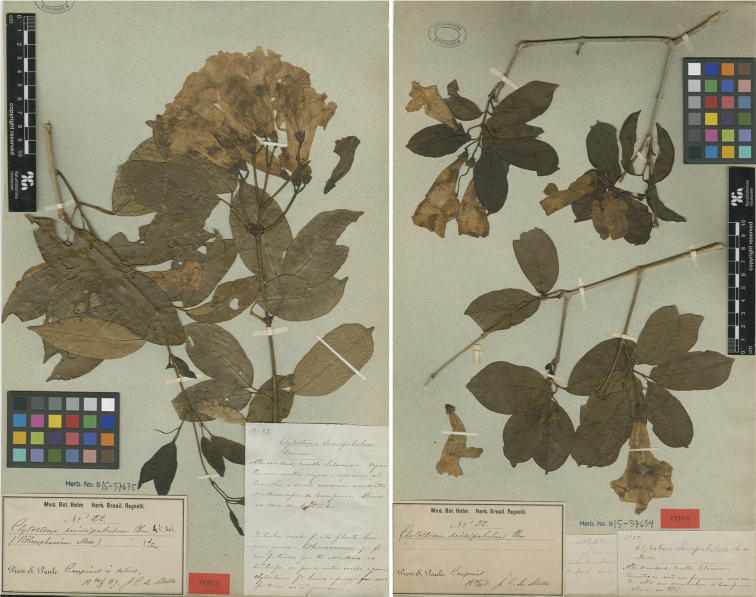
*Clytostoma sciuripabulum* Hovel. S15-37635, and S15-37634 (Neotype). Copyright of images: Naturhistoriska riksmuseet, Stockholm.

Recently, Lohmann (2008: 272 as ‘*sciuripabula*’) transferred *Clytostoma sciuripabulum* to the genus *Bignonia*. Lohmann and Taylor (2014: 422) cited as holotype a specimen non extant at Berlin: Brasil, São Paulo, prope Campinas, 20 IX 1867, *Joaquim Correia de Méllo 22* (B†). The label data provided by Lohmann and Taylor (l.c.) belong to the sample C10021684 (a duplicate donated by S, identified by K.Schumann as *Clytostoma sciuripabulum* Bur.), reproduced in F photo neg. 22132. At S, there are two specimens with the same collection date: S15-37635 with a note written in Portuguese by Correia de Mello, stating that he had coined for this plant the genus *Pithecoxanium*, but it was not used because Miers had previously created the genus *Clytostoma* (Figure 1 – left) and S15-37638.

**Table 1.** *Clytostoma sciuripabulum* - Mello 22, list of syntypes.

Herb. – number	Locality	Date	Phenology	Identification	Other
P608077 Barcode	Bras aust, Sao Paulo, Campinas	27-10-1866	Sterile, with tendrils		Memo written in French
P608079 Barcode	Bras aust, Sao Paulo, Campinas	27-10-1866	Fl + 2 seeds, with tendrils	Clytostoma noterophilum ou tres voisin	
P608078 Barcode	Bras aust, Sao Paulo, Campinas	– (same date in P database)	Sterile, with tendrils		
C10021684 Barcode	Brasil prov Sao Paulo	20-9-1867	Fl, with tendrils	Clytostoma sciuripabulum !Schumann	F photo neg. 22132
S15-37635 Herb. number	Brasil Sao Paulo Campinas in silvis	20-9-1867	Fl, with tendrils	Clytostoma sciuripabulum !Schumann	Memo written in Portuguese
S15-37638 Herb. number	Brasil prov Sao Paulo	20-9-1867	Fl, with tendrils	Clytostoma sciuripabulum	
S15-37634 Herb. number	Bras aust, Sao Paulo, Campinas	10-10-1871	Fl, without tendrils	Clytostoma sciuripabulum	Comments in Portuguese
K449461 Barcode	Bras aust, Sao Paulo, Campinas	Rec. from Herb Hanbury 1/67	Fr, with tendrils	Clytostoma sciuripabulum ex nº	
K449462 Barcode	Bras. merid Campinas	Rec. from Herb Hanbury 3/77	Fl, with tendrils	Clytostoma sciuripabulum	
S-S09-553 Herb. number	Brasil prov Sao Paulo	–	Fruit only	Clytostoma sciuripabulum	

Ulloa Ulloa (2016) detected that the name *Clytostoma sciuripabulum* had been validly published first by Hovelacque (1888: 214). This author published anatomical research about the vegetative organs of Bignoniaceae and other families. Concerning the Bignoniaceae, only the stems and leaves were studied. There is only a brief morphological description of these organs and Hovelacque stated that he had never observed tendrils in *Clytostoma* (page 284). It seems that Hovelacque was not intending to describe a new species, he was only using a name provided most probably by Bureau. Nevertheless, according to Art. 38.1(a), his publication is valid and IPNI (2016) qualified Bureau and Schumann’s name as an isonym; the valid combination cited is: *Bignonia sciuripabulum* (Hovel.) Lohmann (2008: 272).

In the introduction to his work, Hovelacque stated that he used material he cultivated or collected in different regions of France and also material provided largely by the Faculté des Sciences de Lille (LILLE), Institut Botanique de Liége (LG), Bruxelles Botanical Garden (BR) and Muséum de Paris (P).

Information has been requested about *Clytostoma sciuripabulum* from the institutions mentioned by Hovelacque and the response was that there is no material at the herbaria LILLE and LG, while at BR there are only some specimens collected in the 20^th^ century. At the herbarium P, there are 6 specimens collected in the 19^th^ century: P02885030 (Venezuela, Funck & Schlim 962), P02885031 (Paraguay, Hassler 4503), P02885034 (Brazil, Saint Hilaire) and 3 specimens of Mello 22. Since Hovelacque acknowledged his gratitude to Bureau in the introduction to his work and the only collection cited by Bureau and Schumann (1896) is Mello 22, it is assumed that Bureau (author of the specific epithet) had not studied the other samples.

All things stated, no original material studied by Hovelacque is extant, and there is material available for the lectotypification purpose. As a consequence, a neotype should be selected that matches Hovelacque’s description (Art. 9.7). Amongst the specimens of Mello 22 found, listed in Table 1, the only one without tendrils is S15-37634 (Figure 1-right), so it is here designated as the neotype of *Clytostoma sciuripabulum* Hovel.

## Taxonomic treatment

*Bignonia sciuripabulum* (Hovel.) L.G. Lohmann (2008: 272 as ‘*sciuripabula*’). *Arrabidaea sciuripabulum* (Bur.) K. Schumann (1894: 224), *nomen nudum*. *Clytostoma sciuripabulum* Bur. & K. Schum. (1896: 149). Basionym: *Clytostoma sciuripabulum* Hovelacque (1888: 111, 284). **Type**: BRASILIA aust., Sao Paulo, prope Campinas, 10-10-1871 (neotype, designated here, S15-37634, image!).

## References

[B1] Bureau LE Schumann KM (1896) Bignoniaceae in Martius, C.F.P. Flora brasiliensis 8(2): 149.

[B2] Hovelacque M (1888) Recherches sur l’appareil végétatif de Bignoniacées, Rhinanthacées, Orobanchées et Utriculariées. Masson G (Ed.) Paris. Pgs. 111 (adnot. 1), 284. http://gallica.bnf.fr/ark:/12148/bpt6k5654255q

[B3] JSTOR Global Plants (2017) JSTOR Global Plants. https://plants.jstor.org/ [continuously updated]

[B4] JSTOR Global Plants (2017) The International Plant Names Index. http://www.ipni.org/ [continuously updated]

[B5] Lohmann LG (2008) In: Hokche O, Berry PE, Huber O (Eds) Nuevo Catálogo de la Flora Vascular de Venezuela. Fundación Instituto Botánico de Venezuela Dr. Tobías Lasser, Caracas, Venezuela: 272.

[B6] Lohmann LG Taylor CM (2014) A new generic classification of tribe Bignonieae (Bignoniaceae). Ann. Missouri Bot. Gard. 99: 348–489. https://doi.org/10.3417/2003187

[B7] McNeill J Barrie FR Buck WR Demoulin V Greuter DL Hawksworth DL Herendeen PS Knapp S Marhold K Prado J Proud’Homme van Reine WF Smith JF Wiersema JH Turland NJ (Eds) (2012) International Code of Nomenclature for algae, fungi and plants (Melbourne Code): Adopted by the Eighteenth International Botanical Congress, Melbourne, Australia, July 2011. Regnum Vegetabile 154: 1–204. http://www.iapt-taxon.org/nomen/

[B8] Schumann KM (1894) Bignoniaceae in Engler A, Prantl K (Eds) Die Natürlichen Pflanzenfamilien 4(3b): 224.

[B9] Thiers B (2017) Index Herbariorum: A global directory of public herbaria and associated staff. New York Botanical Garden’s Virtual Herbarium, http://sweetgum.nybg.org/science/ih/ [continuously updated]

[B10] Ulloa Ulloa C (2016) In: World Checklist of Bignoniaceae Facilitated by the Royal Botanic Gardens, Kew. Published on the Internet. http://apps.kew.org/wcsp/namedetail.do?name_id=320879

